# Establishment of HPLC Fingerprints for Feiqizhong Tablets and Simultaneous Determination of Fourteen Constituents

**DOI:** 10.1155/2024/7703951

**Published:** 2024-09-18

**Authors:** Ting Yang, Fang Deng, Xuejun Yang, Shunxiang Li

**Affiliations:** ^1^ School of Pharmacy Hunan University of Chinese Medicine, Changsha 410208, China; ^2^ Inspection and Testing Center Hengyang Market Supervision, Hengyang 421001, China; ^3^ Hunan Engineering Technology Research Center for Bioactive Substance Discovery of Chinese Medicine, Changsha 410208, China; ^4^ Hunan Province Sino-US International Joint Research Center for Therapeutic Drugs of Senile Degenerative Diseases, Changsha 410208, China

## Abstract

**Aim:**

To establish the HPLC fingerprints for Feiqizhong tablets and to simultaneously determine fourteen contents in Feiqizhong tablets.

**Methods:**

The analysis of the methanol extract of this drug was performed on a 30°C thermostatic Shimadzu Shim-pack GIST-C18 column (250 mm × 4.6 mm, 5 um), with the mobile phase comprising acetonitrile (*A*) −0.1% phosphoric acid (B) flowing at 1.0 mL/min in a gradient elution manner (0–10 min, 88% A; 10–30 min, 88%–50% A; 30–60 min, 50%–30% A; 60–65 min, 30% A; 65–70 min, 30%–88% A; 70–75 min, 88% A), and the detection wavelength was fixed at 246 nm.

**Results:**

There were one hundred and five common peaks in the HPLC fingerprints of twenty batches of samples with similarities of more than 0.953. Among them, fourteen major active components with a high response value in the chromatogram were selected for quantification, including eupalinolide B, tanshinone IIA, chlorogenic acid, psoralenoside, isopsoralenoside, icariin, amygdalin, caffeic acid, calycosin-7-O–*β*-D-glucoside, salvianolic acid B, quercetin, psoralen, isopsoralen, and bakuchiol. Fourteen constituents showed good linear relationships within their own ranges (*r* ≥ 0.9985), whose average recoveries were 90.71–106.14% with RSD values of 0.23–1.22%.

**Conclusion:**

A rapid and effective method for the separation and detection of components in Feiqizhong tablets is established, which can provide a basis for quality control and quantitative detection.

## 1. Introduction

The current quality standard for Feiqizhong tablets was included in “Pharmaceutical Standard Preparation of Traditional Chinese Medicine Prescription of Ministry of People's Republic of China” (the 10^th^ volume, Standard No. WS3–B-3249-98), and the medicine standards were published in 1993. Inspection items of Feiqizhong tablets include character, identification, and examination. Feiqizhong tablets are a compound preparation of Chinese and Western medicine with complex composition. It is prepared from 9 kinds of medicinal materials, including *Eupatorii Lindleyani Herba*, *Epimedii Folium*, *Salviae Miltiorrhizae Radix et Rhizoma*, *Carthami flos, Fructus Psoraleae*, *Semen Persicae*, *Astragali Radix*, *Vitex Oil*, and Clenbuterol Hydrochloride. Feiqizhong tablets have definite curative effect and are widely used in the clinic. It has the effects of tonifying the kidney and replenishing qi, activating blood circulation and removing blood stasis, and relieving cough and eliminating phlegm. In clinical practice, it is mainly used for pulmonary and renal insufficiency, phlegm obstructing lung, chest tightness and suffocation, frequent dyspnea, cough and expectoration, waist and knee pain, chronic bronchitis, and obstructive emphysema.

The determination of the content of active ingredients in pharmaceutical preparations is very important to ensure the safety and effectiveness of pharmaceuticals. At present, there are no quantitative indicators for the components of Feiqizhong tablets in “Pharmaceutical Standard Preparation of Traditional Chinese Medicine Prescription of Ministry of People's Republic of China (the 17th volume)”. In the existing quantitative studies on Feiqizhong tablets, only icariin [1], psoralen, and isopsoralen [2] have been studied, which are mainly the effective components of epimedium and psoralen. The existing research cannot accurately and effectively control and evaluate the drug quality of Feiqizhong tablets.

Quality control has always been a difficult problem for the development of traditional Chinese medicine and traditional Chinese patent medicine. The most important part of the control of traditional Chinese medicine is to determine the content of its effective ingredients. At present, there are many methods for the determination of drug content, including high-performance liquid chromatography (HPLC) [3–5], thin layer chromatography (TLC) [6], liquid chromatography-mass spectrometry (LC-MS) [7–9], gas chromatography-mass spectrometry (GC-MS) [10], inductively coupled plasma mass spectrometry (ICP-MS) [11], capillary micellar electrokinetic chromatography [12], and infrared spectroscopy [13]. HPLC has the characteristics of high efficiency, fast speed, and stability. Therefore, it is widely used in the qualitative and quantitative analysis of the active components of traditional Chinese medicine and Chinese patent medicine.

In recent years, the quality control methods of comprehensively indicating the quality characteristics of active components in traditional Chinese medicine preparations by fingerprint have become a popular evaluation model [14–17]. However, this method is rarely used in the study of Feiqizhong tablets. The aim of this work was to establish the HPLC fingerprint of Feiqizhong tablets and simultaneously determine the contents of Eupalinolide B, tanshinone IIA, chlorogenic acid, psoralenoside, isopsoralenoside, icariin, amygdalin, caffeic acid, calycosin-7-O–*β*-D-glucoside, salvianolic acid B, quercetin, psoralen, isopsoralen, and bakuchiol, which can provide scientific basis for the quality control of this preparation, to ensure the safety and effectiveness of clinical medication.

## 2. Materials and Methods

### 2.1. Instruments

Quantitative HPLC analysis was performed on Shimadzu LC-20 AT chromatography system equipped with Shimadzu SPD-M20 A detector. The following other instruments were used: XSE205 electronic analytical balance (Mettler Toledo), KQ-500 E ultrasonic extractor (Kunshan Meimei Ultrasonic Instrument Co., Ltd., China), XMTD-4000 Eight-hole electric thermostatic water bath (Beijing Yongguang Medical Instrument Factory, China), and Milli-Q System Ultra-pure water meter (Millipore).

### 2.2. Reagents and Materials

There were 20 batches of Feiqizhong tablets' samples (Guangdong Wanfang Pharmaceutical Co., Ltd., batch numbers: 210904, 210906, 210601, 210901, 201204, 221104, 221001, 210702, 211005, 220203, and 220407; Jiangsu 707 Natural Pharmaceutical Inc., batch numbers: 210302, 210204, 220702, 210302, 200504, 210301, 220402, 220403, and 210323). All of the standard substances, tanshinone IIA (No. 110766–202022, purity: HPLC >99.5%), chlorogenic acid (No. 110753–202119, purity: HPLC >96.10%), icariin (No. 110737–202017, purity: HPLC >94.2%), amygdalin (No. 111637–202111, purity: HPLC >88.20%), caffeic acid (No. 110885–201703, purity: HPLC >99.7%), calycosin-7-O–*β*-D-glucoside (No. 111920–201907, purity: HPLC >96.8%), salvianolic acid B (No. 111562–201917, purity: HPLC >97%), quercetin (No. 100081–201610, purity: HPLC >99.1%), psoralen (No. 110739–201918, purity: HPLC >99.9%), and isopsoralen (No. 110738–202016, purity: HPLC >99.4%) were purchased from the National Institute for Food and Drug Control. Eupalinolide B (No. MUST-21110721, purity: HPLC >96.85%) was purchased from Chengdu Mansit Biotechnology Co., Ltd. (Chengdu China). Isopsoralenoside (No. 250299−202112, purity: HPLC >98.00%) was purchased from Shanghai Hongyong Biotechnology Co., Ltd. (Shanghai China). Bakuchiol (No. CFS202101, purity: HPLC >98.0%) was purchased from Chem Faces (Wuhan China). Psoralenoside (No. 020163–202112, purity: HPLC >98.00%) was purchased from Shanghai Hongyong Biotechnology Co., Ltd. (Shanghai China). The samples were purchased from the drugstore located in Hengyang City, Hunan Province, China. Acetonitrile and methanol are chromatographically pure. Water is deionized distilled water. All other reagents used in this study were of analytical grade and supplied by Sinopharm Chemical Reagent Co., Ltd. (Shanghai, China).

### 2.3. Preparation of Mixed Standard Solution

The salvianolic acid B, chlorogenic acid, affeic acid, calycosin-7-O–*β*-D-glucoside, psoralen, amygdalin, tanshinone IIA, icariin, quercetin, isopsoralen, Eupalinolide B, isopsoralenoside, bakuchiol, and psoralenoside were accurately measured in appropriate quantity and placed in a 10 ml volumetric bottle, dissolved by ultrasound with methanol, and cooled down. The mixed-standard reserve solution was prepared. The concentration of mixed-standard reserve solutions was as follows: *salvianolic acid B* (266.95 *μ*g/mL), *chlorogenic acid* (11.21 *μ*g/mL), *affeic acid* (17.85 *μ*g/mL), *calycosin-*7-O–*β*-D*-glucoside* (20.05 *μ*g/mL), *psoralen* (394.35 *μ*g/mL), *amygdalin* (796.00 *μ*g/mL), *tanshinone IIA* (59.20 *μ*g/mL), *icariin* (125.00 *μ*g/mL), *quercetin* (37.35 *μ*g/mL), *isopsoralen* (493.60 *μ*g/mL), *Eupalinolide B* (123.03 *μ*g/mL), *isopsoralenoside* (131.05 *μ*g/mL), *bakuchiol* (1616.65 *μ*g/mL), and *psoralenoside* (310.30 *μ*g/mL). The mixed-standard reserve solution was reserved in the refrigerator at 0–5°C. The 2.00 ml mixed-standard reserve solution was placed in a volumetric bottle and diluted to 10 ml with methanol, and the mixed standard solution was prepared.

### 2.4. Preparation of Sample Solutions

There were twenty batches of Feiqizhong tablets, each with a batch of ten tablets, which were separately decoated and ground into fine powder. Firstly, 2.0 g of each Feiqizhong tablets' sample powder was accurately weighed and added into a volumetric flask and dilute methanol to 50 mL. Secondly, the mixture was ultrasonicated at 30°C for 30 min. After the mixture was cooled to room temperature, methanol was used to fill the lost weight. Finally, the sample solutions were filtered through 0.22 *μ*m filters before HPLC analysis.

### 2.5. Chromatographic Conditions

The chromatographic column was SHIMADZU Shim-pack GIST-C18 (250 mm × 4.6 mm, 5 *μ*m). The mobile phase was 0.1% phosphoric acid solution (A) acetonitrile (B), gradient elution (0–10 min, 88% A; 10–30 min, 88%–50% A; 30–60 min, 50%–30% A; 60–65 min, 30% A; 65–70 min, 30%–88% A; 70–75 min, 88% A). The flow rate was 1.0 mL/min, the column temperature was 35°C, the detection wavelength was 246 nm, and the sample volume was 10 *μ*L.

## 3. Results

### 3.1. Similarity Evaluation

**Figure 1:**
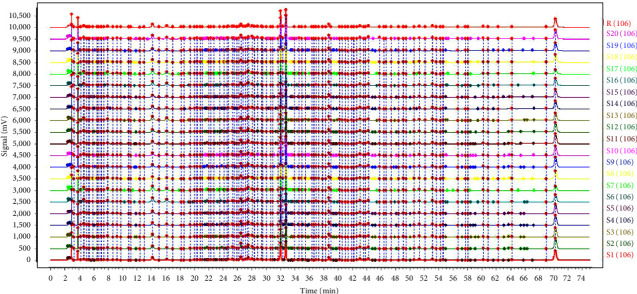
HPLC fingerprint of 20 batches samples. The chromatographic conditions in 2.5.

The fingerprint chromatograph of twenty batches of Feiqizhong tablets were established by the Similarity Evaluation System for the chromatographic fingerprint of (Traditional Chinese medicines) TCMs (Version 2012, Chinese Pharmacopoeia Committee) with the multipoint correction. As presented in Figure 1, the chromatograms were overlaid and aligned, which identified 105 common peaks. As is shown in Table 1, the proposed method's similarity was greater than 0.953, which indicated that twenty batches of samples shared high similarity. The internal quality of the drug was relatively stable.

### 3.2. Chromatographic Peak Identification

**Figure 2:**
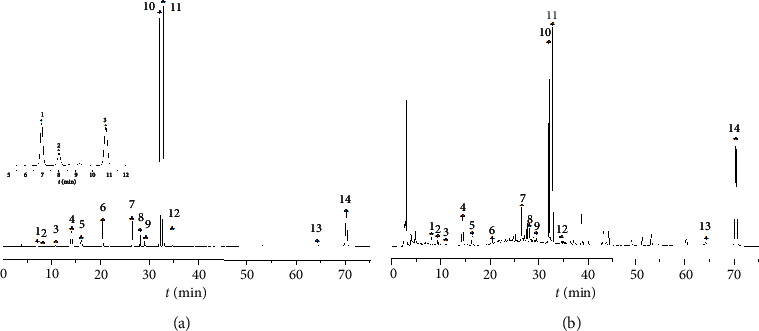
HPLC chromatogram of mixed-standard solution and sample solutions. The chromatographic conditions in 2.5, (a) mixed-standard solution, (b) sample solutions, (1) chlorogenic acid, (2) amygdalin, (3) caffeic acid, (4) psoralenoside, (5) isopsoralenoside, (6) calycosin-7-O–*β*-D-glucoside, (7) salvianolic acid B, (8) icariin, (9) quercetin, (10) psoralen, (11) isopsoralen, (12) Eupalinolide B, (13) tanshinone IIA, and (14) bakuchiol.

By comparing the chromatogram of the sample with that of the reference substances, fourteen common characteristic peaks were not identified from 105 common peaks, and the identification results are shown in Figure 2. The signal-to-noise ratio (SNR) of each characteristic peak was greater than 10, which met the requirement of quantitative analysis. The SNR of peaks 1, 3, 6, 9, 12, and 13 was 10.01, 12.09, 24.92, 47.67, 31.46, and 22.31. The retention time of chromatographic peaks of 14 components in sample solution and mixed-standard solution was the same, and each target peak was completely separated from adjacent peaks.

### 3.3. Method Validation

#### 3.3.1. Calibration Curves

Mixed solutions of chlorogenic acid, amygdalin, caffeic acid, psoralenoside, isopsoralenoside, calycosin-7-O–*β*-D-glucoside, salvianolic acid B, icariin, quercetin, psoralen, isopsoralen, Eupalinolide B, tanshinone IIA, and bakuchiol were prepared with concentrations of 9.02 ug/ml, 259.50 ug/ml, 5.28 ug/ml, 136.30 ug/ml, 59.90 ug/ml, 98.30 ug/ml, 260.00 ug/ml, 107.60 ug/ml, 17.18 ug/ml, 495.00 ug/ml, 612.40 ug/ml, 61.52 ug/ml, 34.78 ug/ml, and 619.60 ug/ml, respectively. The mixed-standard solution of 8 ml, 4 ml, 2 ml, and 0.4 ml was put into each 10 ml volumetric bottle. Methanol was used to supplement the solution, and five linear solutions of different concentrations were prepared. The results (Table 2) showed that fourteen calibration curves represented excellent linearity with high squared correlation coefficient values (*R*^2^ ≥ 0.9991) within the detected range.

#### 3.3.2. Precision

Precision was conducted to evaluate the performance of the instruments. The same sample solution of 10 *μ*L was injected for 6 consecutive times under chromatographic conditions of “Section 2.5.” Precision was evaluated in terms of relative standard deviations (RSD). The RSD values of salvianolic acid B, chlorogenic acid, caffeic acid, calycosin-7-O–*β*-D-glucoside, psoralen, amygdalin, tanshinone IIA, icariin, quercetin, isopsoralen, Eupalinolide B, isopsoralenoside, bakuchiol, and psoralenoside were 1.24%, 0.89%, 0.73%, 1.23%, 1.68%, 1.57%, 1.45%, 1.67%, 1.48%, 1.29%, 0.99%, 1.28%, 1.64%, and 1.75%. The RSD values of fourteen compounds were less than 2.0%, which showed that the precision of the instrument met the experimental requirements.

#### 3.3.3. Repeatability Test

The same batch of emphysema tablet samples (batch no. 210906) was taken to prepare 6 samples of test solution. The peak areas of 14 components were measured by the same chromatography (Section 2.5), and the mass fractions of 14 components were calculated by linear curves. The six samples were analyzed for quantification of fourteen compounds. The repeatability was evaluated in terms of relative standard deviations (RSD) of content of the compounds. The RSD values of salvianolic acid B, chlorogenic acid, caffeic acid, calycosin-7-O–*β*-D-glucoside, psoralen, amygdalin, tanshinone IIA, icariin, quercetin, isopsoralen, eupalinolide B, isopsoralenoside, bakuchiol, and psoralenoside were 1.20%, 0.86%, 0.77%, 1.26%, 1.58%, 0.91%, 1.70%, 1.68%, 1.34%, 1.45%, 0.79%, 1.42%, 1.76%, and 1.81%. The RSD values of fourteen compounds were less than 2.0%, which showed that the experimental method was feasible.

#### 3.3.4. Stability Test

The sample solutions (placed at room temperature) under “Section 2.5” were injected into HPLC at 0, 4, 8, 12, 24, and 48 h for determination, the peak area of salvianolic acid B, chlorogenic acid, caffeic acid, calycosin-7-O–*β*-D-glucoside, psoralen, amygdalin, tanshinone IIA, icariin, quercetin, isopsoralen, Eupalinolide B, isopsoralenoside, bakuchiol, and psoralenoside was recorded, and the RSD values of fourteen components were 1.17%, 1.63%, 1.27%, 0.71%, 1.16%, 1.44%, 0.52%, and 0.71%. The RSD values of fourteen compounds were less than 2.0%, which showed that the test solution was stable at room temperature within 48 h.

#### 3.3.5. Recovery Test

The recovery test used the spiked recovery rate determination method. A sample solution was prepared using 0.1884 g of Feiqizhong tablets (lot number: 220202). Three samples of high level, middle level, and low level were prepared by adding a mixed solution of low, middle, and high concentrations in the sample solution, respectively. Three samples of each level were prepared in parallel. The samples were injected under the chromatographic condition of “Section 2.5.” The calculated recovery rate is shown in Table 3. The average recovery range of the fourteen components was 94.99%–104.93%, and the RSD values range was 0.10%–1.46%. The results illustrated that the proposed method was of good accuracy.

### 3.4. Sample Content Determination

The 2 g sample was accurately weighed from the same batch of the sample, and 3 sample solutions were prepared in parallel according to the method under “Section 2.4.” The samples were injected under the chromatographic conditions of “Section 2.5” for determination, and the content was calculated. The results are shown in Table 4. The content determination results showed that psoralen content in Feiqizhong tablets was the highest. The contents of other components were listed in order from high to low: amygdalin, isopsoralen, psoralen, psoralenoside, salvianolic acid B, Eupalinolide B, isopsoralenoside, icariin, tanshinone IIA, quercetin, caffeic acid, chlorogenic acid, and calycosin-7-O–*β*-D-glucoside.

## 4. Discussion

### 4.1. Extraction Methods' Analysis

In the early stage of the experiment, the effects of extraction solvent (90% methanol, 50% methanol, pure methanol, 0.5% hydrochloric acid solution, and water), extraction time (0.25 h, 0.5 h, 1 h, 1.5 h, and 2 h), and extraction method (ultrasound, heating reflux, and grinding dissolution) on the extraction of medicine components in Feiqizhong tablets were investigated.

It was found that the ultrasonic extraction method extracted more chemical substances than the other two extraction methods, and the operation process was simpler. The separation degree of each extract was higher when methanol was used. When the ultrasonic time was less than 0.5 h, the increase of ultrasonic time was conducive to the extraction of components in Feiqizhong tablets. The extraction gain effect was not obvious when the ultrasonic time was more than 0.5 h. Therefore, methanol was selected as the extraction solvent, and 0.5 h ultrasound was used to extract materials from the Feiqizhong tablets.

### 4.2. Extraction Conditions' Analysis

The organic phase of the mobile phase (methanol, acetonitrile), the water phase of the mobile phase (water, 0.1% phosphoric acid, 0.5% phosphoric acid), and column temperature (20°C, 25°C, 30°C, 35°C, and 40°C) were also investigated to study the separation effect. The results showed that the optimal extraction conditions were 0.1% phosphoric acid in the aqueous phase, acetonitrile in the organic phase, 30°C column temperature, and gradient elution.

The samples were scanned at full wavelength using UV spectra from 210 to 440 nm. The results showed that at the wavelength of 246 nm, the chromatographic information of the tested samples was more comprehensive and fourteen characteristic peaks of the target components all responded strongly. Therefore, the detection wavelength was determined to be 246 nm.

### 4.3. Selection of Detection Index Components

Eupalinolide B is a marker component of *Eupatorii Lindleyani Herba*, which has the effect of eliminating phlegm and cough, clearing heat and detoxifying. Chlorogenic acid and caffeic acid are also important characteristic components of *Eupatorii Lindleyani Herba* [18]. Tanshinone IIA and salvianolic acid B are the marker components of *Salviae Miltiorrhizae Radix et Rhizoma* [19]. Salvianolic acid B has the effect of activating blood circulation, removing blood stasis and activating collaterality. Tanshinone IIA has the effect of vasodilation and has antihypertensive and antithrombotic properties. Psoralenoside, isopsoralenoside, isopsoralen, psoralen, and bakuchiol are the marker components of *Fructus Psoraleae* [20]. Icarii, amygdalin, calycosin-7-O–*β*-D-glucoside, and quercetin are the marker components of *Epimedia Folium*, *Semen Persicae*, *Astragali Radix*, and *Carthami flos*, respectively [21–23]. Among them, isopsoralen, psoralen, and quercetin have anti-inflammatory and antibacterial properties. Eupalinolide B, tanshinone IIA, chlorogenic acid, psoralenoside, isopsoralenoside, icariin, amygdalin, caffeic acid, calycosin-7-O–*β*-D-glucoside, salvianolic acid B, quercetin, psoralen, isopsoralen, and bakuchiol are the 14 components that can effectively identify the main medicinal materials of emphysema tablets and characterize their main effects.

### 4.4. Analysis of Content Determination Results

By analyzing 20 batches of different batches and manufacturers of pulmonary emphysema tablets, including Eupalinolide B, tanshinone IIA, chlorogenic acid, psoralenoside, isopsoralenoside, icariin, amygdalin, caffeic acid, and calycosin-7-O–*β*-D-glucoside, salvianolic acid B, quercetin, psoralen, isopsoralen, and bakuchiol. The preliminary analysis of the content determination results showed that the content of each component in different batches of Feiqizhong tablets was significantly different. The content difference of salvianolic acid B was the highest, ranging from 0.32 to 2.51 mg/g, with a difference of 7.8 times. Due to the relatively fixed preparation method and production process of emphysema tablets, the differences in the content of different components mainly stemmed from fluctuations in raw materials and the level of production process control.

## 5. Conclusion

In this study, HPLC fingerprints of the Feiqizhong tablets were established, and the common peaks were identified. The type and quantity of chemical components contained in the Feiqizhong tablets were reflected comprehensively, and the overall description and evaluation of the quality of the Feiqizhong tablets were realized. A method for the simultaneous determination of 14 characteristic components such as salvianolic acid B, chlorogenic acid, caffeic acid, calycosin-7-O–*β*-D-glucoside, psoralen, amygdalin, tanshinone IIA, icariin, quercetin, isopsoralen, eupalinolide B, isopsoralenoside, bakuchiol, and psoralenoside in the Feiqizhong tablets was established. The method had good precision, repeatability, and stability and could fully reflect the characteristics of raw materials in medicinal materials. This study had an important reference value for the quality control and evaluation of this preparation and provided theoretical support for improving the quality control methods of the Feiqizhong tablets and subsequently improving the quality control standards of the Feiqizhong tablets.

## Figures and Tables

**Table 1:** Similarities of 20 batches samples.

Batch number	Similarity	Batch number	Similarity
210904	0.997	210302	0.953
210906	0.996	200504	0.997
210302	0.985	210301	0.998
210204	0.958	210702	0.997
210601	0.997	220402	0.985
210901	0.997	220403	0.998
201204	0.994	210323	0.997
221104	0.998	211005	0.998
221001	0.997	220203	0.997
220702	0.984	220407	0.997

**Table 2:** Linear relationships of various constituents.

Analytes	Regression equation	Linear range (ug/mL)	*r* ^2^	LOD (ug/mL)	LOQ (*μ*g/mL)
Chlorogenic acid	*y* = 17456*x* + 122.23	0.361 ∼ 9.015	0.9992	0.059	0.198
Amygdalin	*y* = 180.92*x* + 415.11	10.380 ∼ 259.500	1.0000	3.003	10.011
Caffeic acid	*y* = 33310*x* + 191.63	0.211 ∼ 5.280	0.9999	0.039	0.129
Psoralenoside	*y* = 37354*x* + 9952.3	5.452 ∼ 136.300	0.9999	0.239	0.797
Isopsoralenoside	*y* = 38091*x* + 3735.8	2.396 ∼ 59.900	1.0000	0.256	0.855
Calycosin-7-O–*β*-D-glucoside	*y* = 64782*x* + 8902.9	3.932 ∼ 98.300	0.9998	0.059	0.198
Salvianolic acid B	*y* = 12424*x* − 1921.3	10.400 ∼ 260.000	1.0000	0.270	0.901
Icariin	*y* = 13692*x* + 2552.5	4.304 ∼ 107.600	1.0000	0.404	1.346
Quercetin	*y* = 38747*x* + 7744.4	0.687 ∼ 17.180	0.9995	0.086	0.285
Psoralen	*y* = 75933*x* + 99473	19.800 ∼ 495.000	0.9995	0.114	0.379
Isopsoralen	*y* = 66024*x* + 141145	24.492 ∼ 244.920	0.9991	0.134	0.446
Eupalinolide B	*y* = 2252.5*x* − 11.93	2.461 ∼ 61.515	0.9999	0.337	1.123
Tanshinone IIA	*y* = 23451*x* + 3349.1	1.391 ∼ 34.775	0.9998	0.157	0.524
Bakuchiol	*y* = 21219*x* + 58284	24.780 ∼ 619.600	0.9999	0.500	1.668

**Table 3:** Recoveries of fourteen detected compounds.

Analytes	Molecular formula	Average recovery rate/%			RSD/%		
		High-level	Middle-level	Low-level	High-level	Middle-level	Low-level
Chlorogenic acid	C_16_H_18_O_9_	104.93	103.90	101.92	0.45	0.78	1.26
Amygdalin	C_20_H_27_NO_11_	101.99	104.04	103.84	0.47	0.22	1.24
Caffeic acid	C_9_H_8_O_4_	97.27	99.97	99.27	0.38	0.73	0.49
Psoralenoside	C_17_H_18_O_9_	101.19	100.94	99.97	0.30	1.43	1.45
Isopsoralenoside	C_17_H_18_O_9_	98.71	98.39	97.40	0.46	1.46	1.30
Calycosin-7-O–*β*-D-glucoside	C_22_H_22_O_10_	103.04	102.92	104.11	0.35	0.48	1.15
Salvianolic acid B	C_36_H_30_O_16_	104.88	104.07	103.96	0.11	0.09	0.18
Icariin	C_33_H_40_O_15_	99.98	99.41	99.74	0.17	0.12	0.68
Quercetin	C_15_H_10_O_7_	97.84	97.25	97.98	0.26	0.19	0.35
Psoralen	C_11_H_6_O_3_	98.23	98.75	98.47	0.60	0.84	0.39
Isopsoralen	C_11_H_6_O_3_	95.60	94.99	95.21	0.31	0.58	0.56
Eupalinolide B	C_24_H_30_O_9_	98.30	98.85	98.90	0.19	0.10	0.72
Tanshinone IIA	C_19_H_18_O_3_	98.30	98.82	97.90	0.19	0.10	0.72
Bakuchiol	C_18_H_24_O	100.21	100.23	100.43	0.19	0.42	0.83

**Table 4:** Content determination results of various constituents (mg/g, *n* = 3).

Lot number	Salvianolic acid B	Chlorogenic acid	Caffeic acid	Calycosin-7-O–*β*-D-glucoside	Psoralen	Amygdalin	Tanshinone IIA	Icariin	Quercetin	Isopsoralen	Eupalinolide B	Isopsoralenoside	Bakuchiol	Icariin
210904	1.79	0.06	0.08	0.04	2.74	3.52	0.41	0.60	0.20	3.14	1.10	0.56	11.60	1.45
210906	1.72	0.06	0.06	0.03	2.53	3.11	0.39	0.56	0.19	2.91	0.97	0.49	10.92	1.24
210302	1.23	0.04	0.13	0.05	1.65	6.05	0.23	0.59	0.22	1.78	1.14	1.37	8.36	2.18
210204	0.32	0.04	0.12	0.04	1.58	6.40	0.15	0.44	0.15	1.84	0.80	1.14	4.93	2.19
210601	0.92	0.06	0.06	0.06	1.98	3.12	0.41	0.52	0.21	2.79	1.04	0.56	9.17	1.49
210901	1.38	0.07	0.07	0.04	1.85	2.50	0.33	0.50	0.19	2.30	0.93	0.50	9.14	1.13
201204	1.81	0.08	0.09	0.09	2.72	3.32	0.49	0.91	0.27	3.58	1.32	0.80	10.64	1.89
221104	1.88	0.07	0.07	0.04	2.48	3.69	0.39	0.66	0.20	3.10	1.29	0.58	11.61	1.53
221001	1.90	0.07	0.06	0.03	2.29	3.25	0.40	0.60	0.18	2.93	1.17	0.50	10.97	1.29
220702	0.33	0.04	0.13	0.05	1.83	6.89	0.24	0.66	0.24	1.99	1.29	1.49	8.88	2.36
210302	0.34	0.04	0.11	0.05	1.72	6.95	0.16	0.45	0.16	2.00	0.88	1.15	5.17	2.18
200504	1.51	0.07	0.06	0.04	1.83	2.60	0.33	0.51	0.17	2.28	0.97	0.50	9.07	1.10
210301	1.99	0.08	0.07	0.04	2.51	3.71	0.42	0.64	0.20	3.17	1.25	0.59	11.86	1.49
210702	1.88	0.07	0.06	0.03	2.27	3.19	0.40	0.59	0.18	2.90	1.15	0.50	11.15	1.28
220402	1.42	0.04	0.13	0.06	1.83	6.89	0.24	0.65	0.24	1.98	1.28	1.48	9.07	2.37
220403	1.03	0.06	0.06	0.06	1.96	3.61	0.41	0.55	0.21	2.75	1.28	0.57	9.04	1.49
210323	1.42	0.07	0.06	0.04	1.84	2.60	0.33	0.51	0.18	2.29	1.07	0.51	10.91	1.10
211005	1.76	0.06	0.06	0.04	2.21	3.04	0.40	0.61	0.20	2.71	1.29	0.57	11.16	1.34
220203	2.51	0.09	0.07	0.03	2.67	3.58	0.45	0.70	0.21	3.13	1.37	0.68	12.39	1.59
220407	1.86	0.08	0.06	0.05	2.04	2.65	0.37	0.61	0.17	2.49	1.23	0.73	10.42	1.38

## Data Availability

The data from this study are available upon request from the corresponding author.
